# Understanding of perinatal mental health and its psychosocial determinants through Ukrainian women’s experience

**DOI:** 10.18332/ejm/188194

**Published:** 2024-06-05

**Authors:** Nataliia Gusak, Sally Kendall, Olena Nizalova

**Affiliations:** Department of Social Work, National University of KyivMohyla Academy, Kyiv, Ukraine; University of Kent, Kent, United Kingdom

**Keywords:** perinatal mental health, psychosocial determinants, LMIC, Ukraine

## Abstract

**INTRODUCTION:**

Perinatal mental health defines new mothers, their families, and the social, emotional, and cognitive development of their children. The factors contributing to Ukrainian mothers’ mental health are not well-defined in the literature. This study aims to explore how Ukrainian women understand mental health and its psychosocial determinants through their perinatal experience.

**METHODS:**

This qualitative analysis is part of a larger mixed-methods study exploring perinatal mental health in Ukraine. Five online focus groups (n=30) with Ukrainian mothers of children aged 0–5 years were conducted in June–July 2020. The participants were selected from a pool of 1634 women who completed an online questionnaire and agreed to participate in further research. Informed consent was obtained. The data collected from the focus groups were transcribed verbatim and analyzed thematically using Dedoose software.

**RESULTS:**

The study identified two themes. The first theme was: ‘Understanding perinatal mental health through women's experience’, which covers five subthemes. The second theme was ‘Psychosocial determinants of maternal mental health’, which includes six subthemes. Overall, women's feelings of guilt, blame, and shame during their perinatal journey are influenced by socio-cultural factors and can lead to mental health problems and reluctance to seek proper help.

**CONCLUSIONS:**

The study has identified some factors that can contribute to the enhancement of mental health and well-being of mothers in Ukraine during their perinatal journey. Negative emotions such as guilt, blame, and shame can have a significant impact on their ability to seek the necessary support, and should be addressed by midwives and other healthcare professionals.

## INTRODUCTION

The experience of pregnancy, childbirth and becoming a mother is a special time for women full of emotions and changes in daily life. Whilst most of them recognize it as a positive life event, some women might experience perinatal mental health (PMH) problems. PMH refers to a woman’s mental health during pregnancy and the first years after birth, and includes mental illness that existed before pregnancy, developed for the first time, or is greatly exacerbated in the perinatal period^1^.

The prevalence of mental health problems, primarily depression and anxiety, worldwide ranges between 10% during pregnancy and 13% after childbirth^2^. In low- and middle-income countries (LMICs), this is even higher, i.e. 15.6% in the prenatal and 19.8% in the postnatal period^3^. In some LMICs, epidemiological data on PMH is limited and not collected within the official statistics.

The study on mental health in Ukraine showed that one in three Ukrainians experienced at least one mental health problem in their lifetime; depression was prevalent in 6% of the general population (5% regional average in Eastern Europe)^4^. However, there are some studies about PMH among internally displaced females. A 2016 study revealed that internally displaced pregnant women in Ukraine had a 34.8% frequency of post-traumatic stress disorder^5^. Another study showed that pregnant women who were displaced had 3.3 times higher reactive anxiety and 2.6 times higher personal anxiety^6^. There is also evidence suggesting that war-affected populations have increased risks of reactive and personal anxiety, depressive manifestations, autonomic dysfunction, insomnia, and premature termination of pregnancy^7^. Currently, an ongoing study aims to investigate the impact of war on PMH, including anxiety, post-traumatic stress, depression, and birth trauma symptoms^8^.

Numerous studies contributing factors related to PMH in LMICs including poor socio-economic conditions, interpersonal problems, and adverse life events^3^. PMH is significantly associated with multiple socio-economic risk factors, including the young age of mothers^9^, low income, unemployment, low literacy^10^, food insecurity, poor job experience^11^, and poor social support^11,12^. Rates of depression may be increased in settings where women are exposed to multiple risks^11^. Interpersonal problems such as poor marital relationships^12^, lack of partner support^13,14^, poor family support^11,12,15^, and physical and psychological intimate partner violence^12,16,17^, increase vulnerability for PMH problems. Furthermore, evidence also indicated a strong association with previous history of mental disorders^11,12^, unplanned or unwanted pregnancy^15^, becoming overweight or obese during pregnancy^10^, poor infant health and difficulties with breastfeeding, negative obstetric history^12^, and stressful life events^9,18^.

Untreated PMH problems affect mothers, children, and the whole family. Maternal depression during pregnancy increases the risk of having preterm birth and low birth weight^12^. Cook et al.^19^ highlighted that maternal postpartum PTSD is associated with low birth weight and lower rates of breastfeeding. According to Alhusen et al.^10^, increased depressive symptoms, decreased social support, young age, and becoming overweight or obese during pregnancy were significant predictors of non-engagement in favorable health practices during pregnancy.

The affected mothers cannot function properly and as a result, the children’s growth and development may be negatively affected as well including poor infant growth and cognitive development^3^. According to recent research data^20^, first 1001 days (the period from conception to two years) are critical for the development of a child’s healthy brain and their physical, social, and emotional well-being. Evidence is now well developed to show that this period in the growth and development of an infant can be adversely affected by experiences such as deprivation, physical or emotional neglect, alcohol and drug misuse, all forms of abuse, PMH problems, maternal loss among other factors known as Adverse Childhood Experiences^21,22^.

Investment in early intervention for mothers and infants can ensure longer term economic benefits to the healthcare system and to the community, as the evidence is demonstrating that poor health outcomes during the 1001 days will lead to long-term deficits in mental and physical development, education, social and emotional wellbeing, addictive behaviors and entry into the criminal justice system^23^. Supporting women during the critical 1001 days requires an understanding of the many contributing factors to PMH. The aim of this study is to explore how Ukrainian women understand mental health and its psychosocial determinants through their experience in the perinatal period.

## METHODS

### Study design and settings

The qualitative data generated for this study are part of a larger mixed-methods study exploring Perinatal Mental Health in Ukraine (PMHUa-2020). Prior to this study, researchers conducted a 2-day workshop on maternal and infant health in June 2019 in Kyiv to develop strong partnerships with local service providers and experts. This study was conducted among Ukrainian women in their perinatal journey receiving services from non-governmental organizations which agreed to support the study. Data were collected during February–August 2020 by an online survey of five focus groups with women (30 participants), and five focus group and three interviews with professionals (34 participants). The research question addressed in this study is: ‘How do women interpret PMH and its psychosocial determinants through their experience in the perinatal period?’. Ethical approval was obtained from the University of Kent, School of Sociology, Social Policy and Social Research Ethics Committee (SRCEA id 262).

### Participants and recruitment

This mixed-methods study was conducted among mothers of children aged 0–5 years from most oblasts of Ukraine. We focused on the first five years because this is a critical time for child development, especially in developing countries^24^. The inclusion criteria were women who: self-identified as Ukrainians, were able to read and write in Ukrainian, were aged ≥18 years, had children aged 0–5 years, and were willing to participate in the study. The study excluded women who were unable to read and write Ukrainian, were aged <18 years, or had children aged >5 years. Among 1634 women who filled in an online questionnaire, 238 left their contact information for further research. They were approached by the researcher via email with the invitation to participate in an online focus group, 46 signed informed consent forms, and 30 participated in focus groups.

### Data collection

Around six participants are recommended as an optimal size for an online focus group^25,26^, so five online focus groups were conducted in June–July 2020 using Zoom, due to both geographical distances in a country the size of Ukraine and the coronavirus pandemic. Also, ‘Mentimeter’ (an audience engagement platform) has been used to answer anonymously the second question (Table 1). It was designed by the research team based on previous research^27^. The focus group lasted from 90 to 140 minutes. Following the first focus group, the research team reviewed the focus group guide to ensure they were generating good quality data.

**Table 1:** Focus group questions for the study aiming to understand perinatal mental health and its psychosocial determinants through Ukrainian women’s experience, Ukraine, 2020 (N=30)

*No.*	*Questions*
1	Could you introduce yourself and tell us one interesting fact about your being a mother or being pregnant?
2	When we talk about mental health of women during pregnancy and the post-natal period, what comes first to your mind?
3	How would you describe good mental health in a pregnant woman or mother of a small child?
4	What would make you aware that a woman might have mental health difficulties?
5	What things that are going on in life and society could affect mental health and wellbeing of women during pregnancy and the post-natal period?
6	Please, have a look at the women’s stories. Do you remember something similar during your or your friend’s perinatal journey?
7	In your opinion, when do pregnant women and mothers of children aged 0–2 years should seek help for mental health support?
8	Please, have a look at the women’s stories again. Where would they seek help for support in your area?
9	In your opinion, is psychosocial and mental health support available in your area?
10	Did you personally seek help for support for mental health problems during pregnancy or up until your baby turned 2 years old?
11	We would like to hear your ideas of how you could have been better supported whilst experiencing mental health problems throughout pregnancy or the postnatal period. Is there anything specific you would like to have seen?

### Data analysis

**Figure 1:**
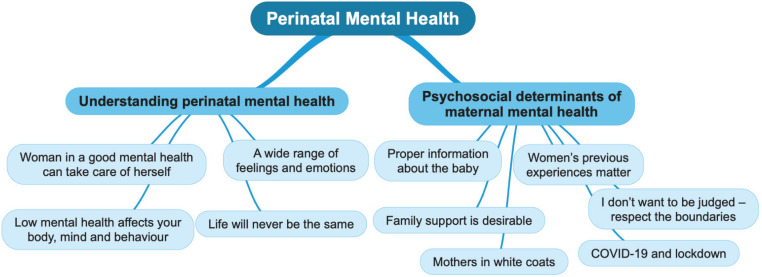
Key themes in understanding perinatal mental health and its psychosocial determinants by Ukrainian women in their perinatal journey, identified from the focus group discussions, Ukraine, 2020 (N=30)

Only seven focus group questions were analyzed for this study including the first six and the last one in order to answer the research question. Audio recordings were transcribed verbatim in Ukrainian, and identification data of participants were removed. Summaries of transcripts were developed both in Ukrainian and English. The transcripts were read and re-read by the researcher, and summaries were reviewed by the research supervisor to confirm the process and contribute to the analysis. Data were analyzed following the thematic analysis and based on the focus group questions^28^. Two overarching and ten subthemes were identified (Figure 1).

## RESULTS

### Characteristics of the participants

Thirty women participated in focus groups. Participants’ age ranged from 23 to 44 years, median age was 33 years. All participants had children: one with 5 children, four with 4 children, three with 3 children, six with 2 children, and sixteen with 1 child. Two out of 30 participants were pregnant. Two women participated from a hospital where they were receiving treatment for complicated pregnancies. Women from 14 out of 25 oblasts of Ukraine participated in the focus groups. Eight of them lived in small towns, the rest in big cities. All women in this study were supported by non-governmental organizations providing services for women in the perinatal period.

### Theme 1: Understanding perinatal mental health through women’s experience

**Figure 2:**
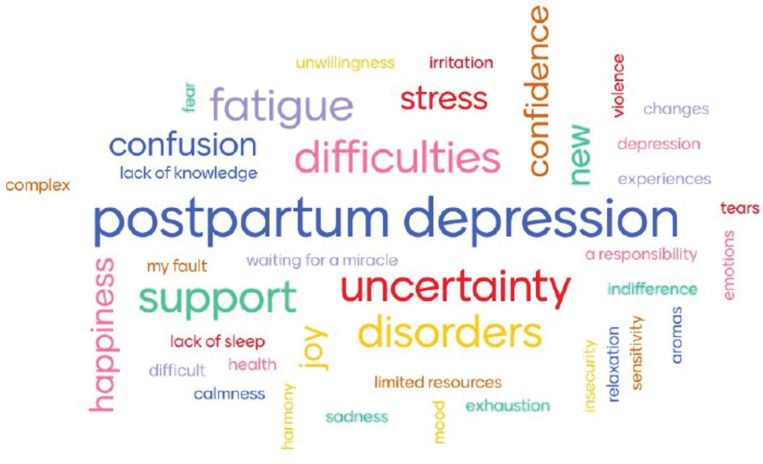
Associations with mental health of women in their perinatal period participating in focus group discussions aiming to understand perinatal mental health and its psychosocial determinants through Ukrainian women’s experience, Ukraine, 2020 (N=30)

Participants associated PMH with such key categories as postpartum depression, difficulties, uncertainty, disorders, and fatigue. Fewer focus group participants mentioned associations such as support, happiness, and confidence (Figure 2). Regarding other associations, many of them could relate to stress in perinatal period, including lack of sleep, limited resources, lack of knowledge, fear, changes, and ‘waiting for a miracle’. The following sub-themes reflect the women’s understanding of their mental health.

*A wide range of feelings and emotions*

Respondents reported a spectrum of different feelings and emotions that women might have, both positive and negative. Numerous participants mentioned that according to the unspoken assumption within Ukrainian society, women are supposed to be happy when they are pregnant or have a baby. Some participants reported that they were happy and confident during pregnancy and after childbirth. One respondent associated PMH with new smells. But most of them highlighted negative feelings and emotions they experienced in the perinatal period, i.e. fear, sadness, fatigue, exhaustion, insecurity, irritability, stress, and guilt (Figure 2). However, those who had multiple pregnancies mentioned that feelings and emotions changed during individual pregnancies and after childbirth, as well as were different with each pregnancy, as one woman describes:

*‘The second child was very desirable for me, but for some reason, I could not cope with my emotions… there was such a feeling of loneliness that I could not cope with …’* (FG1P2)

*Life will never be the same*

Participants who reported negative feelings and emotions, linked these with unrealistic expectations they had before the childbirth. Participants stressed that a woman will never be ready for the significant changes due to childbirth:

*‘… no matter how it is, no matter how ready we are for the pregnancy, for the birth of a child, everything still changes and as it changes in fact, it does not coincide with what it was before the childbirth.’* (FG2P2)

On the one hand, they reported about the stress associated with motherhood and lack of confidence taking care of a baby; however, women do not ask for support with a baby because of the shame:

*‘… The child … cries too much, does not want to sleep – it does not meet any standards and it is very upsetting and this is the feeling when you realize that you cannot cope and are ashamed to ask for help … they may think that you are bad, that you cannot cope, and you will be condemned instead of supported …’* (FG1P4)

On the other hand, their daily life changed significantly both within the family and society. Some participants remembered the strong support they had during pregnancy from relatives, friends, and society. But after the childbirth the situation changed and some of them mentioned being lonely, without expected support from family members and medical staff, as well as being ignored by society:

*‘Pregnancy and what is after are two absolutely different things … pregnancy is really a period when a woman is carried in one’s arm [is being cared for by others], dust particles are blown away, you are a future mother…’* (FG4P2)

*Woman in good mental health can take care of herself*

Talking about their understanding of good mental health, women talked mostly about good psycho-emotional and physical conditions, ability to satisfy their needs and to express emotions, ability to concentrate, to make decisions, to think critically and analyze others’ behavior, to accept and delegate responsibilities, and a desire to realize their own abilities. Additionally, four respondents had their own forums or groups on social media to support women, and two participants founded their own daycare centers for children:

*‘[Has]… the ability to take care of herself, critical analysis of what is said, independent decision-making, the ability to get out of a situation if you get into domestic violence and problems with thinking critically.’* (FG3P4)

*Low mental health affects your body, mind and behavior*

Describing low mental health, participants considered their own experience through physiology, cognition and behavior. A woman with mental health problems might seem exhausted, have sleeping problems and may cry. She may feel sadness, tension, irritability, insecurity, self-blame and guilt. She is an indifferent and aggressive person, who does not care about herself. Respondents mentioned that some women can talk about their difficulties occasionally, but others may keep silent about personal problems. Changes in their behavior might be a signal of mental health difficulties:

*‘… the first is wakefulness … when you stop sleeping and cannot talk about this. … or when she mentioned some difficulties somewhere in conversations or in posts on social networks – self-blame like “I fail” …’* (FG2P1)

### Theme 2: Psychosocial determinants of maternal mental health

Women who participated in focus groups stressed that mental health depends on several determinants related to the baby, woman and her previous experience, family, and society.

*Proper information about the baby*

Participants mentioned several topics related to the baby that might have an impact on PMH. Most of them stressed the importance to be informed about the baby’s health in ethical manner. They also discussed some fear related to baby’s emotions and behavior that should be clearly communicated during pregnancy:

*‘I had a pregnancy with suspicion of genetic abnormalities because … After the screening they recommended to terminate the pregnancy … they all said I was a fool … They said “you understand that the child will be very sick” and all that…’* (FG3P2)

The most intense discussion among women focused on breastfeeding. Participants mentioned several problems with it and none of them was happy with the information and support they received in maternity hospitals. Several women remembered that they got support from breastfeeding consultants they found on social media. Two participants reported about shame in not breastfeeding their babies and said that they would like to receive information that it is acceptable not to breastfeed and that it does not mean that they are ‘bad mothers’:

*‘It was difficult to understand how it is, in the pictures in magazines mother is happy, smiling … and baby is next to her – she is breastfeeding. With my daughter in the first moments – it was just horror, and blood flowed, and milk, and tears, everything in the world … it was very difficult …’* (FG1P2)

*Women’s previous experiences matter*

Among several issues related to women’s experiences, two main topics emerged during focus group discussions. First, difficulties with previous delivery – women reported about their fear to deliver again:

*‘My first delivery was stimulated … three years before the next birth, I had a dot in my head that it was as if I was inferior and not such a woman. I could not give birth. It was psychological… and even in the ward itself.’* (FG4P7)

Second, the unreadiness to have a child because of fear of social isolation, impossibility to realize themselves or to return to work after the maternity leave, and changes in daily life. Two participants mentioned that pregnancy was unwanted because of association of the perinatal period with imprisonment; however, unwanted pregnancy does not mean an unwanted child:

*‘… all three pregnancies were unwanted. For me, this is a radical change in my life … imprisonment … there is no one to communicate with because you sit at home 24 hours alone with children.’* (FG2P2)

*Family support is desirable*

Participants reported that spouse support was very important. Most of them had it during their pregnancy, delivery and after the childbirth. Two respondents said that they got nothing from their partners. In terms of support, their spouses expected them to share responsibility for a baby, to take care of it, to help with daily routine such as cleaning, cooking, and washing up. Variety of thoughts were discussed about expected support from other family members, i.e. from mothers:

*‘My mother always told everyone that I was a wonderful mother, and she was not as wonderful a mother as I was. And it supported me because I realized that I was doing everything right.’* (FG2P2)

*I don’t want to be judged – respect the boundaries*

Almost half of the participants stressed that they do not want to be judged and would like to keep their own boundaries. However, according to the Ukrainian unspoken assumptions, people can intervene into a mother’s privacy:

*‘Our society is very, very often accustomed to interfering in the lives of others in any way. These can be people who are somewhere on the street, on the playground, when it is normal for a person to ask, “why are you pregnant, you already have 2 children running around?”.’* (FG1P1)

Some participants stressed that a mother is responsible for everything and guilty if something is going wrong with a baby. This blame and shame were perfectly described:

*‘In our society, for some reason, as soon as a woman gives birth to a child, she [is] immediately blamed [for] everything. She must do everything she hasn’t done before and be guilty of it.’* (FG2P2)

*Mothers in white coats*

Participants reported that according to unspoken assumptions, a mother should be ideal and cope with everything. And social media add to this expectation by advertising using images of a happy mother with a child nearby – smiling, with good hair, skin, and body shape. Several participants mentioned mothers in ‘white coats’, which makes them feel guilty. This expression of having a white coat is a fairly new phrase in Ukrainian language that means that someone believes that they have perfected the art of life or, in this case, motherhood, and do not hesitate to point out to others what they are doing wrong:

*‘My friend – mother in a white coat – said, “well, it’s your own fault, you baby cannot stay alone because you’re carrying it on your arms” … it’s like this …’* (FG2P3)

*COVID-19 and lockdown*

The impact of pandemic and lockdown have been discussed in relation to delivery and communication with family members. The participants who gave birth during the lockdown mentioned that partner presence during delivery was impossible, even when they wanted to have their partners there. It made them feel stressed:

*‘Delivery was difficult … maternity hospitals were already closed for quarantine and I was not warned that I would give birth alone, well, that is, the stress was very great, because I still wanted a partner birth.’* (FG1P4)

Some participants said that the spouse helped them a lot during the lockdown, and it was supportive for their mental health as well. One participant reported about her mental health problems caused by COVID-19 pandemic and social isolation:

*‘My breakdown happened during the quarantine, a very serious one, after which I even went to the doctor, I was prescribed tranquilizers, because I was already insane…’* (FG4P8)

## DISCUSSION

This qualitative study has explored women’s understanding of PMH and its psychosocial determinants through their experience, in the Ukraine. The women in this study were mainly focused on their feelings and emotions through their perinatal journey, specifically the changes after childbirth. They recognized postnatal depression among possible mental health problems while anxiety, prenatal depression and PTSD were not so evident. Also, they were focused on guilt and shame that lie at the foundation of their mental health problems.

During their perinatal journey, women experience a range of emotions and mood changes which have a significant association with depression and anxiety^29^. Most of the women described their perinatal journey as up-and-down from the pregnancy to the postpartum. Mood swings during the early postpartum period can predict psychopathology up to 14 months after giving birth^29^. Moreover, maternal emotion dysregulation, rather than maternal psychopathology, increases the risk of heightened facial affect synchrony in mother–infant interaction. Some focus-group participants mentioned that it is important to understand these feelings, express them, and develop coping strategies, as the changes in daily routine can be challenging.

During focus group discussions on PMH issues, women were able to identify postpartum depression. However, they did not mention anxiety, severe mental disorders, or PTSD. Previous studies have also shown that while awareness of postnatal depression is high, antenatal depression and anxiety are often underrecognized^30^, but present among Ukrainian displaced mothers, as well as PTSD^5,6,7^.

Previous studies showed that antenatal depression increases over the three trimesters^12^ and maternal depression significantly increases from pregnancy to postpartum^31^. In this study, the journey through women’s experience is accompanied by guilt and shame of not being a good mother, not taking proper care of the baby, not coping with the daily home routine, etc. The sense of life being ‘like a puzzle that is falling apart’ is related to the myriad of negative feelings that stem from feeling like a ‘bad mother’ and having no sense of how to cope or get help with those feelings. As Brassel et al.^31^ highlighted, such feelings may elevate a woman’s vulnerability to postpartum depression and have consequences for later maternal bonding. However, being a ‘good enough mother’ is complex and includes two equally important processes for a child’s healthy cognitive development: 1) the mother must devotedly attend to the infant’s every need; and 2) the mother must gradually allow the baby to experience a need apart from its immediate fulfillment^32^.

In this study, it was found that women associate good or poor PMH with their communication, behavior, physical activity, and ability to take care of themselves. Previous research has shown that pregnant women with poor mental health are less likely to engage in favorable health practices during pregnancy^10^. Moreover, taking out time for oneself at least once a week in the first six months after childbirth may have a positive impact on maternal mental health^13^.

Study results meet the WHO definition of mental health as ‘a state of well-being in which an individual realizes his or her own abilities, can cope with the normal stresses of life, can work productively and is able to contribute to his or her community’^2^. However, the ability to contribute to the community was discussed by women together with a feeling of shame and guilt that may lead to mental health problems.

Regarding the key risk factors for PMH problems, almost all of those covered in Fisher et al.^33^ in their systematic review were mentioned in the current study, including socio-economic disadvantage, unintended pregnancy, being younger, lacking intimate partner empathy and support, experiencing intimate partner violence, and having insufficient emotional and practical support. However, nothing was discussed by participants about being unmarried, having hostile in-laws, and giving birth to a female.

While data about the impact of the pandemic are limited, one woman shared her feeling of social isolation during the COVID-19 pandemic that raises her anxiety. As Cameron et al.^34^ suggested, it could be caused by economic issues and uncertainty about having resources to care for the child.

The study highlights that PMH problems are often caused by guilt and shame stemming from cultural stereotypes and backgrounds. This may explain the limited understanding and knowledge of PMH and its determinants in Ukraine. In Ukraine, perinatal healthcare has traditionally been provided by gynecologists in specialized clinics and maternity hospitals. Mental healthcare, on the other hand, has been mainly provided through expensive inpatient psychiatric hospitals that have been known to have cases of abuse and inhumane conditions for patients. As a result, people with mental health disorders may fear the stigma associated with seeking help.

### Strengths and limitations

The study gathered data directly from women who had either received or not received mental health services during pregnancy and the postpartum period. While most research on this topic relies on data from healthcare professionals or patients, our study focused on women’s expertise and allowed us to identify some barriers to seeking help. However, the study would have been stronger if it had included a more diverse sample of women who did not participate in the online survey. Additionally, we conducted focus groups using Zoom, which made it difficult to interpret non-verbal communication. Nevertheless, this approach allowed us to include women from different geographical locations during the pandemic.

## CONCLUSIONS

Understanding of PMH and its psychosocial determinants, by women’s experiences of pregnancy, childbirth and postpartum, is limited in the Ukraine and focused primarily on mood instability and postpartum depression. The findings indicate that feelings of guilt, blame and shame accompanying women in their perinatal journey are socio-culturally determined and lead to PMH problems, stigmatization and resistance to seeking proper help. The importance of providing relevant support and information about PMH and women’s specific needs during the perinatal journey in the Ukraine, as in other parts of the world, is essential for the future well-being of mothers and their children.

## Data Availability

The data supporting this research are available from the authors on reasonable request.
